# Prior Negative Biopsy, PSA Density, and Anatomic Location Impact Cancer Detection Rate of MRI-Targeted PI-RADS Index Lesions

**DOI:** 10.3390/curroncol31080329

**Published:** 2024-08-01

**Authors:** Ahmad N. Alzubaidi, Amy Zheng, Mohammad Said, Xuanjia Fan, Michael Maidaa, R. Grant Owens, Max Yudovich, Suraj Pursnani, R. Scott Owens, Thomas Stringer, Chad R. Tracy, Jay D. Raman

**Affiliations:** 1 Department of Urology, Penn State Milton S. Hershey Medical Center, Hershey, PA 17033, USA; analzubaidi@gmail.com (A.N.A.);; 2 Pennsylvania State College of Medicine, Hershey, PA 17033, USA; 3 Department of Urology, University of Iowa Hospitals and Clinics, Iowa City, IA 52242, USArowens@uiowa.edu (R.G.O.); chad-tracy@uiowa.edu (C.R.T.); 4 Department of Urology, University of Florida College of Medicine, Gainesville, FL 32611, USA; 5 UPMC Central PA Hospital, Camphill, PA 17011, USA; owensrs@upmc.edu

**Keywords:** fusion prostate biopsy, PI-RADS, magnetic resonance imaging, PSA density

## Abstract

Background: MRI fusion prostate biopsy has improved the detection of clinically significant prostate cancer (CSC). Continued refinements in predicting the pre-biopsy probability of CSC are essential for optimal patient counseling. We investigated potential factors related to improved cancer detection rates (CDR) of CSC in patients with PI-RADS ≥ 3 lesions. Methods: The pathology of 980 index lesions in 980 patients sampled by transrectal mpMRI-targeted prostate biopsy across four medical centers between 2017–2020 was reviewed. PI-RADS lesion distribution included 291 PI-RADS-5, 374 PI-RADS-4, and 315 PI-RADS-3. We compared CDR of index PI-RADS ≥ 3 lesions based on location (TZ) vs. (PZ), PSA density (PSAD), and history of prior negative conventional transrectal ultrasound-guided biopsy (TRUS). Results: Mean age, PSA, prostate volume, and level of prior negative TRUS biopsy were 66 years (43–90), 7.82 ng/dL (5.6–11.2), 54 cm^3^ (12–173), and 456/980 (46.5%), respectively. Higher PSAD, no prior history of negative TRUS biopsy, and PZ lesions were associated with higher CDR. Stratified CDR highlighted significant variance across subgroups. CDR for a PI-RADS-5 score, PZ lesion with PSAD ≥ 0.15, and prior negative biopsy was 77%. Conversely, the CDR rate for a PI-RADS-4 score, TZ lesion with PSAD < 0.15, and prior negative biopsy was significantly lower at 14%. Conclusions: For index PI-RADS ≥ 3 lesions, CDR varied significantly based on location, prior history of negative TRUS biopsy, and PSAD. Such considerations are critical when counseling on the merits and potential yield of prostate needle biopsy.

## 1. Introduction

Prostate cancer (PCa) is the most commonly diagnosed male malignancy worldwide. In the United States, PCa incidence increased by 3% annually from 2014 through 2019 after almost 20 years of decline. Over the last year, the United States reported approximately 290,000 cases of PCa with an estimated 35,000 cancer-related deaths [1]. Additionally, although it often has an indolent course, the proportion of prostate cancer diagnosed at an advanced stage has increased from 3.9% to 8.2% over the past decade [2]. 

Men with clinical suspicion of prostate cancer (with elevated PSA and/or abnormal DRE) typically undergo a transrectal (TRUS) or transperineal ultrasound-guided biopsy of the prostate during which 10 to 12 systematic cores are obtained. The transrectal ultrasound-guided approach was previously the gold standard for cancer detection when clinical suspicion is present, but it has been shown to be susceptible to underdiagnosis, grade misclassification, and complications [3,4]. 

Over the last decade, multiparametric magnetic resonance imaging (mpMRI) has been increasingly incorporated into prostate imaging and diagnostic evaluation. mpMRI combines anatomic (T2W phase) and functional assessment (diffusion-weighted imaging and apparent diffusion coefficient maps and dynamic contrast-enhanced) imaging to provide an objective assessment for risk of prostate cancer. This technology has been used with fusion prostate biopsy and has increasingly become the standard of care with improved detection of clinically significant prostate cancer (CSC; Grade Group ≥ 2) [5,6].

In 2012, after the introduction of mpMRI, the American College of Radiology (ACR) introduced a new standard reporting system named the Prostate Imaging Reporting and Data System (PI-RADS) [7], specifically pertaining to the characterization of prostate lesions. A second edition (v2.0) was released in 2016, which was further updated in 2019 and titled version (v2.1) [8,9]. It has since gained popularity among radiologists as a standardized reporting system for interpreting and dictating prostate MRI and gained widespread acceptance as a standard method of diagnosis for suspicion of clinically significant prostate cancer. The PI-RADS scoring system risk-stratifies lesions into five categories based on the size and appearance of the lesion. The categories range from PI-RADS-1 (CSC is highly unlikely) to PI-RADS-3 (CSC is equivocal) and PI-RADS-5 (CSC is highly likely). It does not factor in lesion location and any other patient-related characteristics [10]. The PRECISION trial, a multicenter international randomized controlled trial conducted in 2018 and published in the New England Journal of Medicine, randomized 500 biopsy-naïve patients with clinical suspicion for PCa to undergo mpMRI, with or without targeted biopsy, or standard TRUS biopsy. Men in the MRI group only underwent targeted biopsy if mpMRI showed a lesion with PI-RADS 3 or higher. The study concluded that in biopsy-naïve patients, PI-RADS evaluation with mpMRI could assist 28% to avoid unnecessary biopsy, as well as increase the detection of Gleason Grade (GG) ≥ 2 cancer when MRI-targeted biopsy was used for PI-RADS 3–5 lesions (38% vs. 26% with standard biopsy, *p* = 0.005) [11]. This was similar to previous findings from the PROMIS trial, which were published prior to the PIRADS system, where mpMRI allowed 27% of patients to avoid biopsy with 5% fewer clinically insignificant cancer detected [12]. 

Although notable improvements are observed when compared to conventional prostate biopsy, the existing literature reports variability in diagnostic yield overall and CSC [13,14]. Continued refinements in predicting the pre-biopsy probability of CSC are, therefore, essential for optimal patient counseling. Here, we aim to investigate clinical and radiographic factors that may impact the yield of cancer detection of CSC for PI-RADS ≥ 3 lesions. We hypothesize that lesion location, PSA density, and prior negative TRUS biopsy history may significantly impact cancer detection. Such information may be readily translated into urological practice to ensure informed decision-making for patients considering prostate biopsy.

## 2. Materials and Methods

Consecutive men undergoing MRI-guided prostate biopsy between 2017 and 2020 across 4 different medical centers (3 academic, 1 community) for suspected prostate cancer (elevated PSA and/or abnormal digital rectal examination) with PI-RADS ≥ 3 lesions were included. 

### 2.1. MRI Protocol and Biopsies

In general, MRI images were acquired using a 3-Tesla magnet with annotation of lesions performed using the PI-RADS v2 guideline T2 weighted, diffusion-weighted (DWI), and dynamic contrast-enhanced (DCE) images. The volume (cm^3^) of each region of interest was calculated using a rectangle polygon model after manually measuring the height, width, and length of each lesion. Scans were reviewed and interpreted by fellowship-trained, board-certified radiologists in conjunction with urologists. 

Fusion biopsies were performed by a sub-specialized urologist at each of the 4 respective institutions using a transrectal approach. All biopsies utilized commercially available software for image registration with ultrasound segmentation via the bidimensional ultrasound probe and rendering of a three-dimensional ultrasound volume. Data acquired by ultrasound and MRI are fused together with alignment and a minimum of two biopsies were obtained from index lesions.

### 2.2. Cohort of Analysis

The cohort included 1054 patients with an index lesion (PI-RADS score of ≥3) on multiparametric MRI. Patients with anterior index lesions (n = 74) were excluded due to low numbers across subgroups. With such criteria, a final evaluable cohort of 980 index lesions in 980 patients was established. Index PI-RADS ≥ 3 lesions were further stratified based on location (transitional zone (TZ) vs. peripheral zone (PZ)), PSA density (cutoff of 0.15 ng/mL/cm^3^), and history of prior negative conventional TRUS biopsy. 

### 2.3. Statistical Analysis

Calculations of CDR were performed using R with subset and mean functions using no additional packages. We used the Wilcoxon rank-sum, Kruskal–Wallis tests, Chi-square, and Student *t*-test where applicable. Significance was set at a *p*-value of 0.05.

## 3. Results

Table 1 summarizes the clinical, radiographic, and pathologic characteristics of our cohort. Median patient age and PSA were 66 years (43–90) and 7.8 mg/dL (5.6–11.2), respectively. Approximately 50% of men in our study (456 of 980) had a prior negative biopsy. On MRI, PI-RADS index lesion distribution included 291 PI-RADS 5, 374 PI-RADS 4, and 315 PI-RADS 3. with calculated median prostate volume of 54 cm^3^ (12–173). PZ index lesions were found in 58.7% (575/980) of patients, while TZ index lesions were found in 41.3% (405/980).

Overall, CSC was detected in 346 lesions (35%). More specifically, CSC was detected in 164 lesions (56%), 133 lesions (36%), and 49 lesions (16%) of PI-RADS 5, PI-RADS 4, and PI-RADS 3 lesions, respectively. In aggregate, PZ lesions were more likely to harbor CSC compared to TZ lesions (42.9% vs. 24.4%, *p* > 0.001). Higher CSC was seen in those with higher PSAD above vs. below cutoff 0.15 (51.7% vs. 24.5%, *p* < 0.001). Additionally, those without a history of prior negative TRUS biopsy had higher rates of CSC (36% vs. 32%, *p* < 0.001).

Within the PZ subgroup, lesions were more likely to be classified as a PI-RADS 5 (64% (185/291) vs. PI-RADS 3 48% (151/315), *p* < 0.001). Conversely, PI-RADS 3 lesions were more likely to be found in a TZ location compared to PI-RADS 5 lesions (52% (164/315) vs. 36% (106/291), respectively, *p* < 0.001).

With respect to PSAD, PI-RADS 5 lesions had a greater percentage of occurring in the high PSAD density group (>0.15) compared to those in the PI-RADS 3 group (58% (170/291) vs. 35% (110/315), respectively, *p* < 0.001). Finally, patients with PI-RADS 5 and PI-RADS 3 were similarly likely to have had a history of prior negative TRUS biopsy (44% (128/291) vs. 45% (141/315) respectively, *p* > 0.05).

Table 2 summarizes the CDR as stratified by PSA density, lesion location, and biopsy history. Notably, significant variance in CDR was observed across subgroups. In particular, the highest CDR was seen in the cohort for a PI-RADS 5 score, PZ lesion in patients with PSAD ≥ 0.15, and prior negative biopsy was 77%. Conversely, the CDR rate for a PI-RADS 4 score, TZ lesion, with PSAD < 0.15, and prior negative biopsy was significantly lower at 14% (*p* < 0.001).

## 4. Discussion

In this study, we evaluated potential factors related to improved cancer detection rates of clinically significant cancer in patients with index PI-RADS ≥ 3 lesions. MRI-targeted prostate biopsy has been shown to improve the detection rate of clinically significant prostate cancer compared to conventional transrectal ultrasound-guided biopsy [6,8,15]. However, surgeon experience, number of targeted biopsy cores, radiologic interpretation, and software systems have collectively contributed to variability in cancer detection rates [14,16]. 

Overall, our cancer detection rates were lower for higher PI-RADS lesions compared to those of the PRECISION trial. CDRs from the PRECISION trial versus our study were 83% vs. 56% for PI-RADS 5, 60% vs. 36% for PI-RADS 4, and 12% vs. 16% for PI-RADS 3 lesions [12]. We believe these observations are critical for iterative feedback at a site-specific level to understand and improve on factors contributing to lower CDR. Nonetheless, we observed that our cohort followed similar patterns in which the percentage of CSC was highest among patients with PI-RADS 5, with a subsequent decline across different PI-RADS classifications.

Our experience across four medical institutions revealed significant discrepancies in cancer detection rates with respect to location, PSAD, and history of prior negative TRUS biopsy. Lesion location had the highest impact on CDR across PI-RADS subgroups. In aggregate, PZ lesions were more likely to harbor CSC compared to TZ lesions (42.9% vs. 24.4%, *p* > 0.001), the highest detection rate occurring in patients with PZ lesions, PSAD > 0.15, and prior negative TRUS biopsy at 77%. The next highest observed CDR was 70% within patients with PZ lesions, PSAD < 0.15, and no prior biopsy. Our results differ from those of a recent study of 263 U.S. veteran patients, which noted that the location of the lesion was not statistically associated with CSC in PI-RADS 3 lesions [17]. In contrast, lesion location was noted to be associated with PI-RADS scoring by a German population study that retrospectively reviewed MRIs of 293 patients. Mahjoub et al. suggested different PI-RADS size cut-offs based on location, with smaller and more defined separation of CSC found in the PZ compared to TZ [18]. Future optimization of PI-RADS could address location variations by accounting for gland heterogeneity, benign prostate hyperplasia, and size criterion [19,20]. Until then, given that most prostate cancers occur in the PZ, clinicians should consider the importance of location, and thus clinical suspicion of CSC in PZ lesions, in assessing the need for biopsy. 

Conversely, the lowest CDRs occurred with PI-RADS 3 and 4 patients with a history of prior negative TRUS and PSAD < 0.15 at 3% and 14%, respectively. This observation adds to the debate on whether biopsy is indicated for PI-RADS scoring with a minority of CSC in the context of wide variation and low positive predictive value of PI-RADS scoring across medical centers [21]. It is generally accepted that PI-RADS 1 and 2 scores are considered likely benign and do not require additional testing. Likewise, PI-RADS 4 and 5 scores are likely associated with CSC and biopsy should be recommended. For equivocal scoring, decision-making is more ambiguous. Biopsy is reasonably indicated if there is continued clinical suspicion for CSC. Yet, close active surveillance is also a reasonable decision for patients who would prefer to avoid biopsy and are able to adhere to monitoring protocols [22]. In a prospective study of 723 men with MRI-visible prostate lesions, a combination of MRI-targeted biopsy and standard systematic biopsy was suggested to be beneficial in eliminating the risk of missing any CSC in PI-RADS 3 or 4 lesions [3]. Minimal changes in CDR occurred for the PI-RADS 5 group, suggesting that MRI-targeted biopsy alone may be sufficient for high-grade lesions [22]. However, the value of systematic biopsy allowed for significant additional detection of 7.5% and 8.0% for the PI-RADS 3 and 4 groups, respectively, compared to MRI-targeted biopsy alone, which yielded 17.2% and 35.8% of CSC, respectively. In contrast, in a study of 92 biopsied PI-RADS 3 lesions by Liddell et al., a low risk of prostate cancer (6.5%) was reported and, therefore, the authors supported ongoing surveillance [23]. Such variability in CDR has made determining the significance and management of seemingly low-risk lesions difficult. 

PSAD, which is obtained by the total PSA (ng/mL) divided by the prostate volume, may serve as an adjunct in cancer detection of ambiguous PI-RADS lesions and has been associated with more aggressive prostate cancer long before the advent of MRI fusion biopsy [24]. Similar to our results, Natale et al. found that PSAD was a significant predictor of CSC in multivariate analyses, with patients with PSAD > 0.15 being five times more likely to have clinically significant disease compared to PSAD < 0.15 [17]. Other studies demonstrated that incorporation of PSAD resulted in higher CDR than PI-RADS alone (50.0–66.7% vs. 48%) for PI-RADS ≥ 3 and a 20% reduction in unnecessary biopsies for indeterminate PI-RAD lesions [25,26]. Contrary to most recent findings, some studies revealed the combination of methods held no significant improvement in diagnostic performance, though this may be dependent on lesion location [27,28]. Despite inconsistent conclusions in the literature, our study supports the use of MRI as a powerful tool in the detection of clinically significant prostate cancer and the impact of PSAD on CDR, where PSAD > 0.15 has significantly higher CDR for all lesions regardless of PI-RADS score. Upcoming predictive models with the integration of PSAD, among other biomarkers and patient features, with PI-RADS have shown promising results by significantly and efficiently reducing unnecessary prostate biopsies within risk thresholds of >10–20% [29,30].

Several limitations exist within our study. There may have been patient-related and prostate-specific covariates that we did not stratify for or identify, e.g., family history of prostate cancer, due to the study’s retrospective nature. Results were collected from four different medical centers, including a community hospital, across the country to account for variability and broaden generalizability. Additionally, there may be variation in PI-RADS lesion classification among interpreting radiologists across institutions, though interrater agreement has been suggested to be sufficient [31]. The number of cores per lesion was also taken at the discretion of the provider, which may have led to the under-detection of CSC in this sample [16]. Nonetheless, the impact of this study shows how real-world results can significantly differ compared to data emerging from trial settings, and thus, using practical data may improve patient counseling on prostate biopsy yield. Further investigation on CDR across more institutions may provide additional insights on applicability for future risk calculation.

## 5. Conclusions

In patients with PI-RADS ≥ 3 lesions, CDR varied significantly based on location, prior history of negative TRUS biopsy, and PSA density. Higher PSA density, no prior history of negative TRUS biopsy, and PZ lesions were associated with higher CDR. Utilization of these factors can improve risk stratification for CSC and therefore develop appropriate guidelines for counseling patients on their candidacy for prostate needle biopsy and prostate cancer management.

## Figures and Tables

**Table 1:** Patient and tumor baseline characteristics (n = 980 index lesions).

**Patient Characteristics**	**Median and IQR or Frequency (%)**
Age at biopsy (years)	66 (61–71)
PSA (ng/dL)	7.82 (5.6–11.2)
Prostate Volume (cm^3^)	54 (40–79)
PSA Density (ng/dL^3^)	0.13 (0.09–0.21)
Prior Negative TRUS Biopsy	
Yes	456 (47%)
No	524 (53%)
**Lesion Characteristics**	
PI-RADS Score	
3	315 (32.1%)
4	374 (38.1%)
5	291 (29.7%)
**Lesion Location**	
Peripheral Zone	575 (58.7%)
Transition Zone	405 (41.3%)

**Table 2:** Percentage of clinically significant cancer detection rates stratified by PI-RADS lesion score, tumor location, PSAD, and history of prior negative transrectal ultrasound-guided biopsy.

PI-RADS 5	CDR % 56% (164/291)	PI-RADS 4	CDR % 36% (133/374)	PI-RADS 3	CDR % 16% (49/315)
PZ	63 (117/185)	PZ	41 (97/239)	PZ	22 (33/151)
*PSAD* ≥ *0.15*	70 (77/110)	*PSAD* ≥ *0.15*	53 (48/91)	*PSAD* ≥ *0.15*	30 (18/61)
*Prior Negative Bx*	77 (49/64)	*Prior Negative Bx*	57 (32/56)	*Prior Negative Bx*	39 (12/31)
*No Prior Bx*	61 (28/46)	*No Prior Bx*	46 (16/35)	*No Prior Bx*	20 (6/30)
*PSAD* < *0.15*	53 (40/75)	*PSAD* < *0.15*	33 (49/148)	*PSAD* < *0.15*	16 (15/90)
*Prior Negative Bx*	29 (9/31)	*Prior Negative Bx*	26 (16/62)	*Prior Negative Bx*	10 (3/30)
*No Prior Bx*	70 (31/44)	*No Prior Bx*	38 (33/86)	*No Prior Bx*	20 (12/60)
TZ	44 (47/106)	TZ	26 (36/136)	TZ	10 (16/164)
*PSAD* ≥ *0.15*	55 (33/60)	*PSAD* ≥ *0.15*	43 (24/56)	*PSAD* ≥ *0.15*	20 (10/49)
*Prior Negative Bx*	67 (10/15)	*Prior Negative Bx*	48 (12/25)	*Prior Negative Bx*	25 (2/8)
*No Prior Bx*	51 (23/45)	*No Prior Bx*	39 (12/31)	*No Prior Bx*	20 (8/41)
*PSAD* < *0.15*	30 (14/46)	*PSAD* < *0.15*	15 (12/80)	*PSAD* < *0.15*	5 (6/115)
*Prior Negative Bx*	22 (4/18)	*Prior Negative Bx*	14 (6/44)	*Prior Negative Bx*	3 (2/72)
*No Prior Bx*	36 (10/28)	*No Prior Bx*	17 (6/36)	*No Prior Bx*	9 (4/43)

## Data Availability

Reviewed and available via standard protocol.
